# Copper and Cobalt Ions Released from Metal Oxide Nanoparticles Trigger Skin Sensitization

**DOI:** 10.3389/fphar.2021.627781

**Published:** 2021-02-19

**Authors:** Sung-Hyun Kim, Jin Hee Lee, Kikyung Jung, Jun-Young Yang, Hyo-Sook Shin, Jeong Pyo Lee, Jayoung Jeong, Jae-Ho Oh, Jong Kwon Lee

**Affiliations:** Division of Toxicological Research, National Institute of Food and Drug Safety Evaluation, Ministry of Food and Drug Safety, Osong, South Korea

**Keywords:** skin sensitization, alternative test, KeratinoSens^TM^, LLNA, dissolving nanoparticles, nanoparticles, copper, cobalt

## Abstract

Human skins are exposed to nanomaterials in everyday life from various sources such as nanomaterial-containing cosmetics, air pollutions, and industrial nanomaterials. Nanomaterials comprising metal haptens raises concerns about the skin sensitization to nanomaterials. In this study, we evaluated the skin sensitization of nanomaterials comparing metal haptens *in vivo* and *in vitro*. We selected five metal oxide NPs, containing copper oxide, cobalt monoxide, cobalt oxide, nickel oxide, or titanium oxide, and two types of metal chlorides (CoCl_2_ and CuCl_2_), to compare the skin sensitization abilities between NPs and the constituent metals. The materials were applied to KeratinoSens^TM^ cells for imitated skin-environment setting, and luciferase induction and cytotoxicity were evaluated at 48 h post-incubation. In addition, the response of metal oxide NPs was confirmed in lymph node of BALB/C mice via an *in vivo* method. The results showed that CuO and CoO NPs induce a similar pattern of positive luciferase induction and cytotoxicity compared to the respective metal chlorides; Co_3_O_4_, NiO, and TiO_2_ induced no such response. Collectively, the results implied fast-dissolving metal oxide (CuO and CoO) NPs release their metal ion, inducing skin sensitization. However, further investigations are required to elucidate the mechanism underlying NP-induced skin sensitization. Based on ion chelation data, metal ion release was confirmed as the major “factor” for skin sensitization.

## Introduction

Metal oxide nanoparticles (NPs) constitute one of the major types of nanomaterials (NMs) that are used in industrial, biomedical, and cosmetic applications. With an increase in the number and production volume of NPs, concerns about their toxicity have increased exponentially in the recent years. While the major NP-exposure pathways include inhalation, ingestion, and absorption into the skin, the latter can cause lesions such as local inflammation, contact allergy, and skin sensitization (Oberdörster et al., 2005; Maynard and Kuempel, 2005). With an exponential increase in the commercialization of NPs in cosmetics and relevant safety concerns, evaluation of NP safety has become important (Katz et al., 2015). In recent cosmetic tests, the importance of alternative test methods considering animal welfare and the 3R principles has been emphasized (Rusche, 2003; Kaluzhny et al., 2011). However, since these guidelines are based on chemical substances, development of alternative test methods reflecting the properties of nanomaterials are also imperative.

The physicochemical properties of NPs form the key determinants of its toxic potential (Donaldson et al., 2013; Braakhuis et al., 2014). NPs, in most cases, are minimally soluble under normal physiological conditions; however, some have been shown to be soluble in certain media, such as lysosomal fluid (Cho et al., 2012; Cho et al., 2013). Dissolution of such NMs causes toxicity different from that caused by NMs that do not dissolve well, possibly due to the released ions. According to Cho et al., 2012, the toxicity of fast-dissolving metal oxide NPs is closely related to the intrinsic toxicity of its constituent metal ions.

Current knowledge regarding the chemical and biological mechanisms associated with skin sensitization has been summarized in the form of an adverse outcome pathway (starting with the molecular initiating event, through intermediate events, to the adverse effect), namely allergic contact dermatitis (OECD, 2018a). The first key event involves the initial covalent reaction of electrophilic chemicals in the irritant with nucleophilic thiol and primary amines in skin proteins. The second key event occurs within the keratinocytes and includes inflammatory responses as well as changes in gene expression associated with specific cell signaling pathways, such as the antioxidant/electrophile response element (ARE)-dependent pathways (OECD, 2014). The ARE-Nrf2 Luciferase KeratinoSens^TM^ test, representing the second key event, can discriminate between skin sensitizers and non-sensitizers under the United Nations Globally Harmonized System of Classification and Labelling of Chemicals (OECD, 2018a).

Park et al., 2011, had reported titanium oxide NPs to not induce skin sensitization in mice, as per a local lymph node BrdU-enzyme-linked immunosorbent assay. In contrast, gold NPs had been shown to bind non covalently to proteins and affect the immune system (Yoshioka et al., 2017). In addition, the NPs may release free chemicals with skin sensitization properties (Dwivedi et al., 2011; Dykman and Khlebtsov, 2017). However, there is still little information on the possible skin sensitization of NPs.

This study aimed to evaluate the skin sensitization potential of metal oxide NPs using the ARE-Nrf2 Luciferase KeratinoSens^TM^ assay and LLNA-FCM assay. In addition, the effect of release of ions from metal oxide NPs on skin sensitization were investigated in a dose-dependent manner.

## Materials and Methods

### NPs and Metal Chlorides

CoO, Co_3_O_4_, CuO, and TiO_2_ NPs were purchased from Nanostructured and Amorphous Materials (Houston, TX, United States). NiO NPs were purchased from US-Nano (Houston, TX, United States). Metal chlorides (CoCl_2_, CuCl_2_; Sigma-Aldrich, St Louis, MO, United States) were used for testing the constituent metal ions. Their primary size was confirmed by transmission electron microscopy (JEM-1200EX II, JEOL, Tokyo, Japan). The hydrodynamic size, polydispersity, and zeta potential of the NPs were measured using a Zetasizer Nano ZS instrument (Malvern Instruments, Malvern, United Kingdom), in different vehicles, including distilled water (DW), Dulbecco’s Modified Eagle’s Medium (DMEM; GIBCO, Grand Island, NY, United States) containing 1% heat-inactivated fetal bovine serum (FBS; GIBCO) for KeratinoSens^TM^ assay, and LLNA: BrdU-FCM assay working solution (DMF solution containing 3% mouse serum). The levels of endotoxin were evaluated using an Endpoint Chromogenic Limulus Amoebocyte Lysate assay (Cambrex Corporation, Walkersville, MD, United States).

### Metal Oxide NPs Dissolution Assay

The dissolution of NPs was measured in artificial lysosomal fluid (ALF, pH 5.5) or phosphate buffered saline (PBS, pH 7.4), as previously described (Jeong et al., 2015, 2016). Briefly, NPs were dispersed in each medium at 100 μg/ml and incubated for 48 h at room temperature. Centrifugation was performed thrice at 15,000 × g for 30 min to collect the NP-free supernatant, and absence of NPs was confirmed by dynamic light scattering (DLS) analysis using a Zetasizer Nano ZS (Malvern). Concentration of metal ions in the supernatant was measured using inductively coupled plasma optical emission spectroscopy (ICP-OES; 700-ES, Varian Inc., United States). Solubility was calculated as the percentage of dissolved metal concentration regarding the initial mass of metal in the NP suspension.

### Cell Culture and Treatment of KeratinoSens^TM^ with NPs

A transgenic cell line with stable insertion of the luciferase reporter gene under the control of ARE-element KeratinoSens^TM^ was obtained from Givaudan Suisse SA (Vernier, Switzerland). The cells were cultured in DMEM supplemented with 10% FBS and 0.5 mg/ml Geneticin (G418; Sigma-Aldrich). KeratinoSens^TM^ cells were sub-cultured every 3–4 days at 80–90% confluence for a maximum of 25 passages. For the experiments, KeratinoSens^TM^ cells were seeded into 96-well plates at a density of 1 × 10^4^ cells/well, the medium was replaced with fresh medium (DMEM supplemented with 1% FBS), and eventually incubated in a humidified atmosphere of 5% CO_2_ at 37°C.

The NP suspensions in media were prepared by modifying the previously described method (Jeong et al., 2018). Briefly, the NP stock solution was dispersed in DW at a concentration of 200 mM and sonicated at 40 kHz with 100 W output power for 10 min in a bath-type sonicator (Saehan-Sonic, Seoul, South Korea). Then, DMEM supplemented with 1% FBS was added to different working concentrations (0.98–2000 µM). Then KeratinoSens^TM^ cells were treated with NPs from a concentration of 2000 µM to serial dilution. Since CoO and Co_3_O_4_ NPs have the same constituent elements, the ion concentration was calculated for comparison and treated as the constituent element ratio. If metal chloride occurs, each cell was treated by converting the ion concentration to the constituent element ratio.

### Luciferase Induction and Cytotoxicity Assay

To assess the induction of luciferase activity in KeratinoSens^TM^ cells, the latter were seeded into 96-well plates at a density of 1 × 10^4^ cells/well and incubated overnight to approximately 80% confluence. The cells were washed thrice with pre-warmed DPBS (Gibco) followed by adding fresh medium containing test materials (0.98–2000 µM) and incubation for 48 h. Luciferase activity was measured using the ONE-Glo^TM^ luciferase assay kit (Promega). Luminescence intensity of each sample was measured using a luminometer (Promega) and multi-microplate reader (Synergy 2, BioTek, Winooski, VT, United States). Luciferase induction was calculated based on the luminescence values of the vehicle control and blank.

For cell viability test, briefly, KeratinoSens^TM^ cells were seeded into 96-well plates at a density of 1 × 10^4^ cells/well and incubated overnight to reach approximately 80% confluence. The cells were washed once with pre-warmed DPBS (Gibco), followed by adding fresh medium containing test materials (0.98–2000 µM) and incubation for 48 h. Cell viability was measured by a thiazolyl blue tetrazolium bromide (3-(4,5-dimethylthiazol-2-yl)-2,5-diphenyl-tetrazolium bromide) assay (Promega, Madison, WI, United States). To exclude colorimetric interference from NPs, present in the cells, the supernatant was transferred into clear 96-well plates and absorbance measured at 570 nm with a microplate reader (Tecan, Männedorf, Switzerland). Cell viability (%) was calculated based on the optical density of vehicle control and blank.

### Treatment of KeratinoSens^TM^ Cells with Solubilized and Chelated Copper and Cobalt Ions

To evaluate the role of dissolved metal ions in cytotoxicity and sensitization, KeratinoSens^TM^ cells were treated with solubilized metal ions followed by chelation, as described in our previous study, with slight modification (Jeong et al., 2018). Briefly, the stock solution of copper and cobalt chlorides in DW were incubated with Chelex 100 beads (Sigma-Aldrich) twice at 1:10 ratio (v/v) and shaken for 8 h at room temperature (26 ± 2°C). The beads were then removed by centrifugation at 15,000 × g for 30 min. The collected supernatant was diluted to the working concentration (2 mM) with cell culture medium and applied to KeratinoSens^TM^ cells.

### *In Vivo* Skin Sensitization Assay: LLNA-FCM

Female BALB/C mice (7 weeks old, specific pathogen free) were purchased from ORIENT BIO Inc (Korea) and acclimated for at least 7 days before the experiments. The animals were kept at an animal facility in the Korea Ministry of Food and Drug Safety (MFDS). They were housed at a temperature of 22 ± 3°C and relative humidity of 30–70%. The study was approved by the Institutional Animal Care and Use Committee (IACUC) (2018, Approval NO. MFDS-18-145). On day 1, 2, and 3, 25 μL of the dispersed NP suspension, vehicle, and positive control (25% hexyl cinnamic aldehyde in AOO) were applied to the dorsal skin of each ear at the same time-point. Briefly, the NP stock solution was dispersed in DW at a concentration and sonicated at 40 kHz with 100 W output power for 10 min in a bath-type sonicator (Saehan-Sonic). After dispersing the nanoparticle stock solution, 3% mouse serum was used as a dispersant and sonicated for 10 min to increase the dispersion efficiency. Then the DMF solution was added at different working concentrations (Low dose: 25, Mid dose: 50, and High dose: 100 %) for each NPs. NP suspensions were prepared fresh daily before application. On day 5, the mice were intraperitoneally injected with 100 μL of BrdU solution (20 mg/ml). On day 6, all mice were sacrificed, and their auricular lymph nodes excised. The latter were then mashed with a spatula to prepare lymph node cells (LNCs). Isolated LNCs were counted using a hemocytometer after staining with trypan blue. The counted LNCs (1.5 × 10^6^ cells/mL) were prepared according to the protocol provided in the kit. Thereafter, viable LNCs were counted and 10,000 gated cells were analyzed using BD FACS Calibur^TM^ flow cytometry (BD Biosciences), as described (Jung et al., 2010; Ahn et al., 2016). Stimulation index (SI) values were calculated using the formula described in OECD test guideline 442B guideline. If the SI values were 2.7 or above, the test substance was classified as sensitizers.

### Histological Procedures for Mouse Ear Tissue Treated with NPs

Mouse ear tissue was fixed with 10% neutral buffed formalin for 24 h, followed by routine histological procedures. The tissue was stained lightly with eosin, which provided contrast from the metal oxide NPs. The NP aggregates in ear tissue were visualized by optical microscopy (Leica, Germany).

### Comparison Between the Component Ions of Highly Soluble NPs by LLNA-FCM Assay

Constituent element ions were calculated from the concentrations of CoO and CuO (which are fast-dissolving NPs), as done previously, to prepare a suspension of constituent ions using corresponding metal chlorides. The concentrations of CoCl_2_, CuCl_2_, metal chlorides, used were the same ion molar concentration as the three concentrations of CoO and CuO nanoparticles tested previously (25, 50 and 100%). The LLNA-FCM assay was performed by treating animals via the same procedure as the described test method for NPs.

### Statistical Analysis

Data of *in vitro* assay are expressed as mean ± SD (n = 6). LLNA: BrdU-FCM data are expressed as mean ± SD (n = 4). Statistical analysis was performed via one-way analysis of variance (ANOVA). Post hoc Tukey’s pairwise comparison was performed for comparing between groups. Results were prepared using GraphPad Prism ver.7.0 (GraphPad Software, San Diego, CA). A *p* value less than 0.05 was considered statistically significant.

## Results

### Physicochemical Properties of the Metal Oxide NPs

**FIGURE 1:**
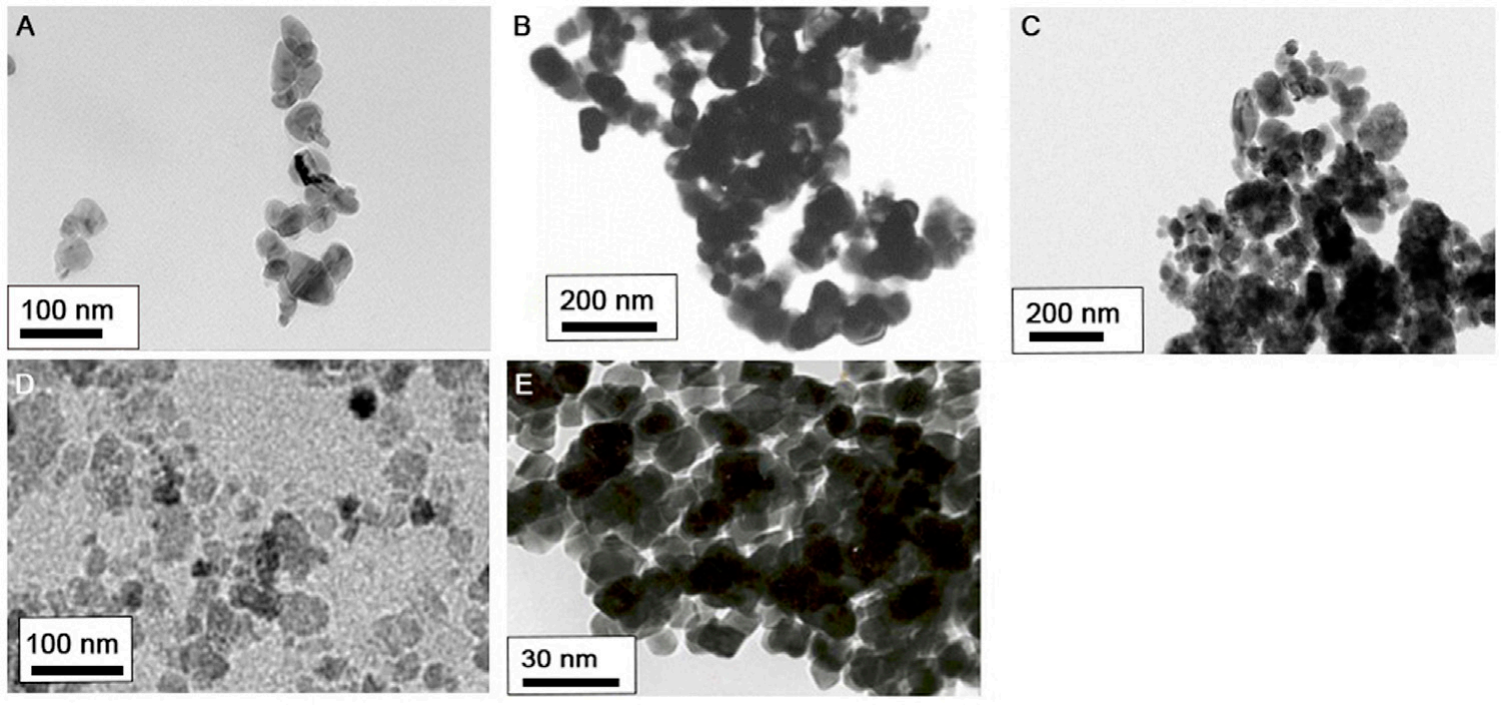
Morphology of five metal oxide NPs observed by transmission electron microscopy (TEM): **(A)** CoO, **(B)** Co_3_O_4_, **(C)** CuO, **(D)** NiO, and **(E)** TiO_2_ NPs.

Transmission electron microscopy images of the five metal oxide NPs used in this study are shown in Figure 1. Average sizes of the different NPs were < 100 nm, like that of NPs provided by manufacturers. Physicochemical properties of the five metal oxide NPs are summarized in Table 1. Hydrodynamic size analysis revealed them all to be agglomerated, based on their primary size (nm). Measurement of zeta potential showed all NPs to be positively charged in DW and negatively charged in DMEM, with zeta potentials of −23 to −32 mV. The Limulus Amebocyte Lysate test showed all metal oxide NPs to have endotoxin levels lower than the limit of detection (0.1 U/mL). The dissolution test showed incubation of CoO and CuO NPs in artificial lysosomal fluid (pH 5.5) for 48 h to result in dissolution of over 77.7%, whereas Co_3_O_4_, NiO, and TiO_2_ NPs had dissolution of 0.2, 2.0, and 0.3%, respectively. However, all NPs dispersed in PBS showed less than 5.4% dissolution.

**TABLE 1:** Physicochemical characterization of the five metal oxide NPs.

NPs	CoO	Co_3_O_4_	CuO	NiO	TiO_2_
Primary size (nm)	43.60 ± 7.94	41.12 ± 12.06	47.80 ± 13.24	19.10 ± 5.97	12.81 ± 2.70
Hydrodynamic size (d, nm) in					
DW	251.8 ± 6.1	294.2 ± 37.2	261.3 ± 11.1	303.5 ± 12.0	491.2 ± 73.8
working solution (DMEM)^a^	403.3 ± 11.1	336.8 ± 45.3	307.1 ± 19.3	276.6 ± 62.1	486.0 ± 14.7
working solution (DMF)^b^	492.9 ± 13.3	499.7 ± 74.4	349.9 ± 7.2	490.9 ± 37.1	793.8 ± 63.3
Polydispersity (PDI) in					
DW	0.45 ± 0.07	0.44 ± 0.16	0.18 ± 0.07	0.32 ± 0.05	0.52 ± 0.28
working solution (DMEM)^a^	0.32 ± 0.04	0.53 ± 0.12	0.36 ± 0.05	0.50 ± 0.11	0.53 ± 0.07
working solution (DMF)^b^	0.39 ± 0.06	0.61 ± 0.19	0.30 ± 0.10	0.42 ± 0.10	0.31 ± 0.03
Zeta potential (mV) in					
DW	17.38 ± 0.55	22.77 ± 4.19	15.33 ± 2.17	33.22 ± 1.80	13.43 ± 0.31
working solution (DMEM)^a^	−26.42 ± 1.10	−28.70 ± 1.13	−28.00 ± 1.20	−23.50 ± 1.87	−24.60 ± 1.19
working solution (DMF)^b^	−18.22 ± 0.46	−33.50 ± 1.59	−26.48 ± 0.57	−32.86 ± 0.90	−34.10 ± 1.00
Solubility (%) in					
ALF (pH 5.5)	77.7	0.2	88.4	2.0	0.3
PBS (pH 7.4)	5.4	0.1	0.1	1.8	0.2
Molecular weight (g/mol)	74.93	240.8	79.5	74.7	79.6
Purity (%)	99.5	100	100	99	99.7
Endotoxin (U/mL)	<0.1	<0.1	<0.1	<0.1	<0.1

DW, distilled water; ALF, artificial lysosomal fluid; PBS, phosphate-buffered saline. Data were expressed as mean ± SD from six independent experiments.

^a^ DW stock (1%) + DMEM containing 1% FBS, condition.

^b^ DW stock (10%) + DMF containing 3% inactivated mice serum, condition.

### Evaluation of NPs-Induced Sensitization in the KeratinoSens^TM^ Assay

**FIGURE 2:**
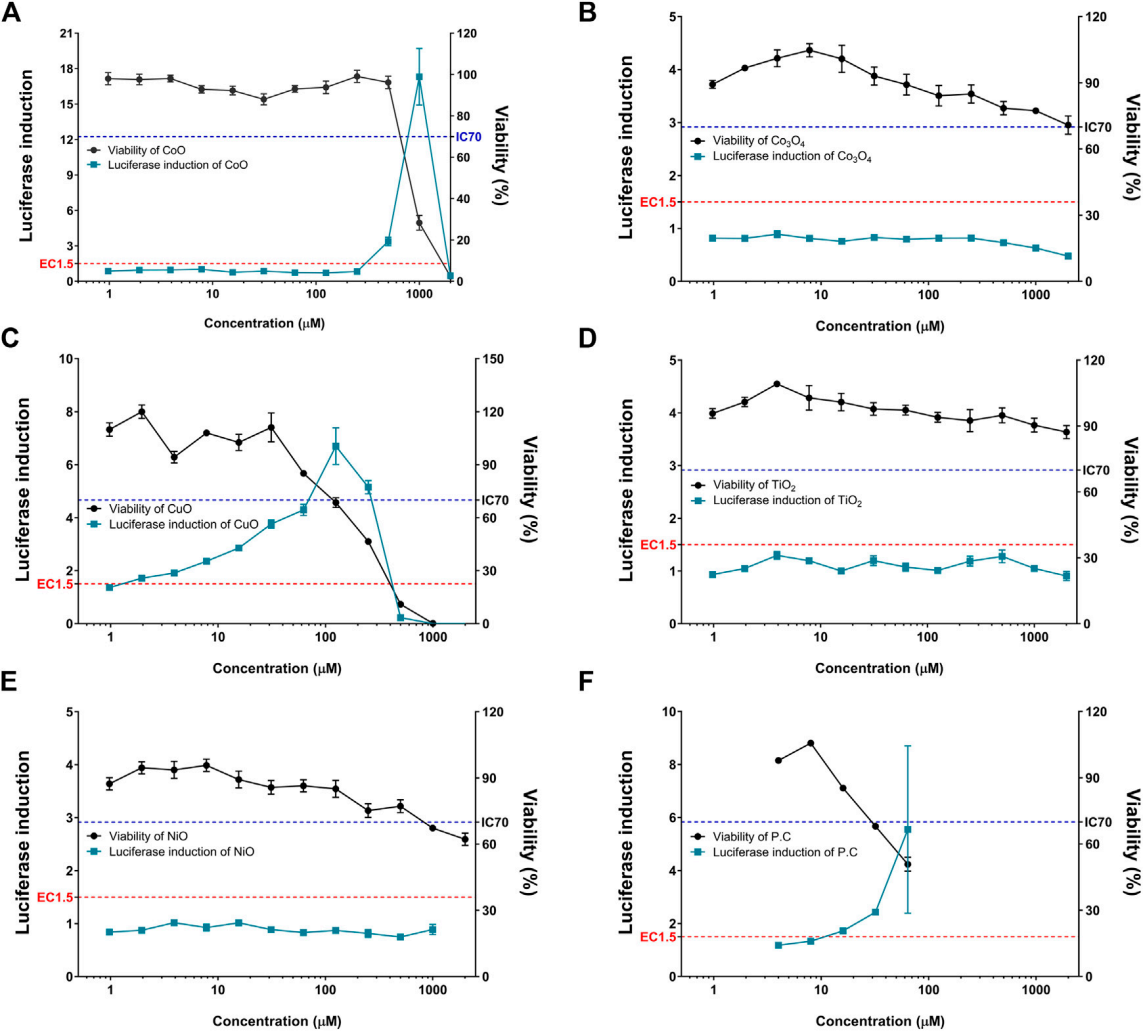
Induction of luciferase activity (green squares) and cell viability (black rounds) in the KeratinoSens^TM^ assay. KeratinoSens^TM^ cells were treated with five metal oxide NPs: **(A)** CoO, **(B)** Co_3_O_4_, **(C)** CuO, **(D)** NiO, and **(E)** TiO_2_ NPs. **(F)** Positive control (cinnamic aldehyde, 4–64 µM) was tested in parallel. Data are expressed as mean ± standard deviation values (n = 6).

The five metal oxide NPs were assessed for their skin sensitization potential using the KeratinoSens^TM^ assay; the data are shown in Table 2 and Figure 2. CuO and CoO NPs-induced activity of the luciferase reporter by over 1.5-fold, suggesting their ability to cause skin sensitization. The other NPs did not increase luciferase activity in the KeratinoSens^TM^ assay. The EC_1.5_ value for CuO and CoO NPs was 1.38 and 316.57 µM respectively, classifying them as sensitizers, whereas the values were >1,000 µM for the remaining NPs, classifying them as non-sensitizers.

**TABLE 2:** KeratinoSens^TM^ assay results with the five metal oxide NPs.

NPs	CAS RN	Physical form	KeratinoSens^TM^ assay results				
			Imax	EC_1.5_ (µM)	Cell viability (%)^a^	IC_50_ (µM)	Classification
CoO	1307-96-6	Solid	17.32	316.57	>70	841.19	Positive
Co_3_O_4_	1308-06-1	Solid	1.07		>70	>2000	Negative
CuO	1317-38-0	Solid	6.70	1.38	>70	122.74	Positive
NiO	1313-99-1	Solid	1.05		>70	>2000	Negative
TiO_2_	13463-67-7	Solid	1.30		>70	>2000	Negative

^a^ Cell viability (%) at EC_1.5_.

### Comparison Between Highly Soluble Metal Oxide (CoO and CuO) NPs and Corresponding Metal Ions

**FIGURE 3:**
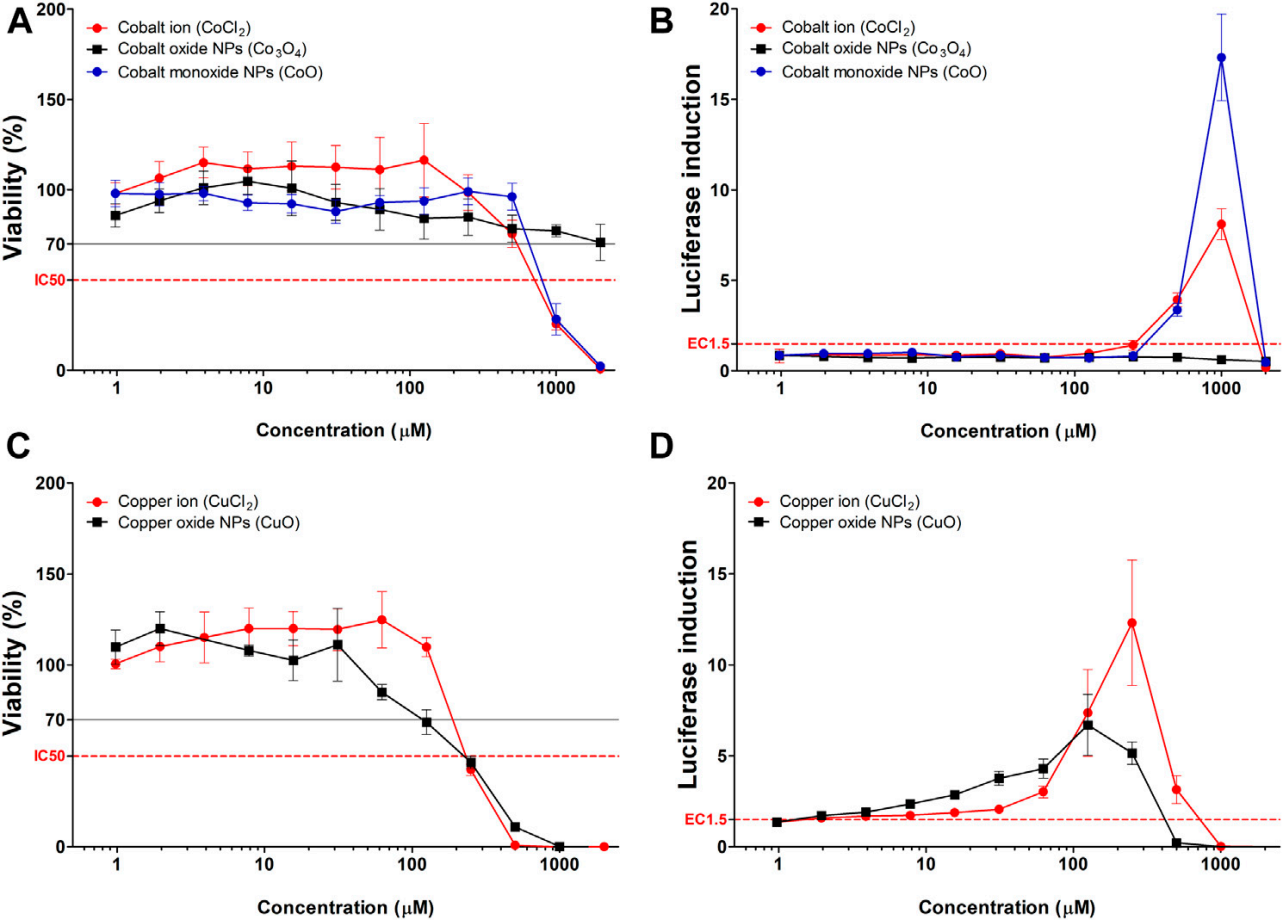
Comparison of luciferase induction assay results between highly soluble metal oxide NPs and their component metal ions. **(A)** Viability and **(B)** luciferase induction CoO, Co_3_O_4_ NPs, and CoCl_2_. **(C)** Viability and **(D)** luciferase induction CuO NPs and CuCl_2_. Data are expressed as mean ± standard deviation values (n = 6).

CoO and CuO NPs were selected based on elemental analysis (ICP-OES) data for studying the effect of NPs with fast-dissolving ability and comparison of the same with corresponding metal ions. Cytotoxicity and luciferase induction in KeratinoSens^TM^ cells were measured using CoCl_2_ and CuCl_2_, which are ionic forms of the nanoparticles (Figure 3). Results of the ion-dose treatment of Cobalt and Copper metal chlorides and treatment of CoO and CuO NPs showed similar dose-dependent features in the two analyses.

### Differential Luciferase Induction After Chelation of Metal Chlorides

**FIGURE 4:**
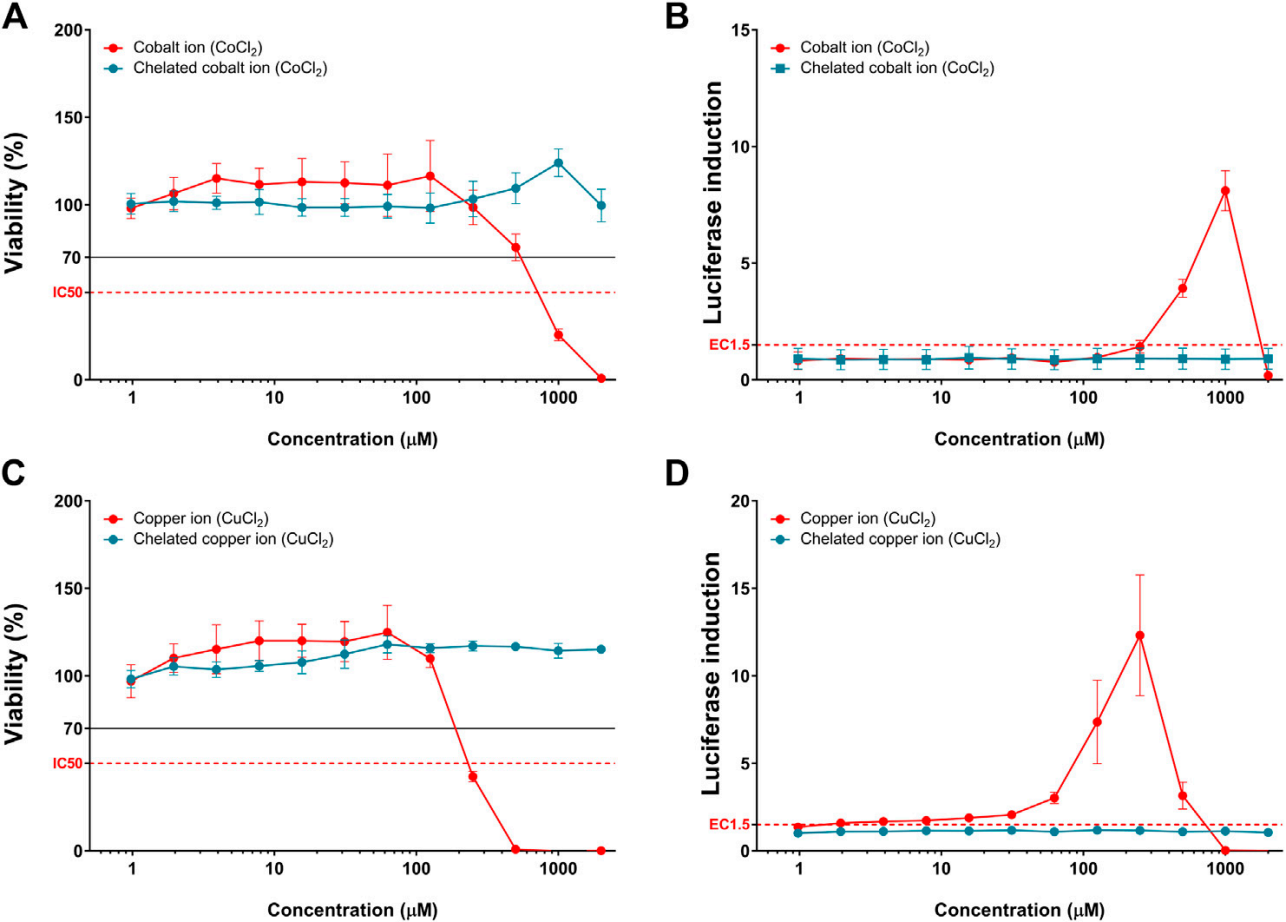
Comparison of luciferase induction and cytotoxicity results between the chelated and non-chelated metal chlorides. **(A)** Viability and **(B)** luciferase induction of cobalt chloride (CoCl_2_). **(C)** Viability and **(D)** luciferase induction of copper chloride (CuCl_2_). Data are expressed as mean ± standard deviation values (n = 6).

The chelation of copper and cobalt chlorides showed dramatic recovery of cytotoxicity, along with non-sensitization results, compared to that with non-chelated metal chlorides (Figure 4). Although metal chlorides showed significant luciferase induction and cytotoxicity in KeratinoSens^TM^ cells, chelation of metals showed no fold induction and cytotoxicity at the doses tested.

### Evaluation of the Five Metal Oxide NPs Using LLNA-FCM Assay

**FIGURE 5:**
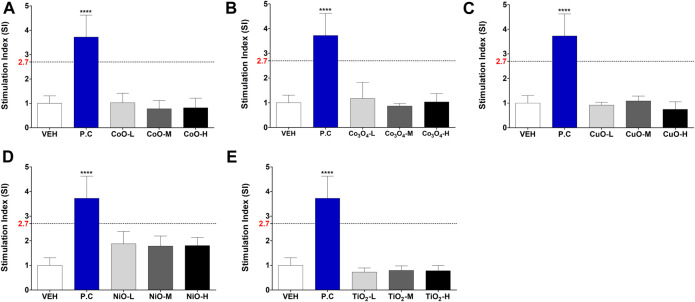
Induction of stimulation index (SI) in the LLNA: BrdU-FCM assay. Animals were administered five metal oxide NPs: **(A)** CoO, **(B)** Co_3_O_4_, **(C)** CuO, **(D)** NiO, and **(E)** TiO_2_ NPs. Data are expressed as mean ± standard deviation values (n = 4). Significance vs. vehicle control: ****p < 0.0001.

Metal oxide NPs were assessed for their skin sensitization potential using the LLNA: BrdU-FCM assay, data shown in Figure 5. The SI was obtained by flow cytometry, and for all metal oxide NPs, SI value was less than 2.7.

### Observation of CuO NP Aggregates in the Stratum Corneum

**FIGURE 6:**
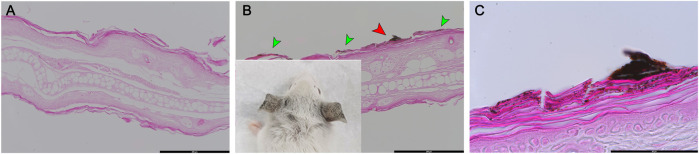
Histologic examination of eosin-stained ear skin tissue after exposure of mice to copper oxide NPs. **(A)** Vehicle control (DMF), **(B)** and **(C)** treated with CuO NPs. CuO NPs accumulated in the stratum corneum (green arrow). **(C)** is a high-magnification image of the red arrow portion in **(B)**. Scale bars: **(A)** 200 μm, **(B)** 200 μm, **(C)** 50 µm.

To visualize the NPs penetration, mouse ear tissues were stained lightly with eosin, which provided contrast from the dark NPs (Figure 6). CuO NP-exposed mouse ear showed large aggregates in the stratum corneum (Figures 6B,C). Upon measurement of the approximate size of the particles, agglomerates of nanoparticles 400–1,000 nm were observed.

### Differential Skin Sensitization Potential of NPs and Component Metal Ions per LLAN:-BrdU-FCM Assay

**FIGURE 7:**
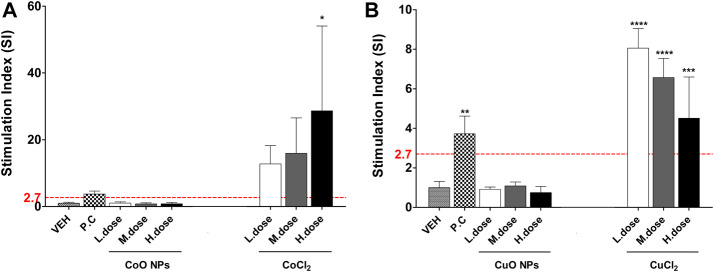
The SI value in LLNA-FCM assay due to metal oxide NPs and component metal ions (Cobalt and copper). Animals were treated with: **(A)** Cobalt-containing compound, and **(B)** Copper-containing compound. Data are expressed as mean ± standard deviation values (n = 4). Significance vs. vehicle control: ****p < 0.0001.

The SI results of NPs and metal chlorides are presented in Figure 7. SI value of the metal oxide NPs was less than 2.7. CoCl_2_ and CuCl_2_ metal chlorides showed higher SI value than metal oxide (CoO and CuO) NPs, respectively.

## Discussion

This study was conducted to evaluate the skin sensitization potential of metal oxide NPs using the ARE-Nrf2 Luciferase KeratinoSens^TM^ assay and LLNA: BrdU-FCM assay. These assays were based on the second and fourth key events of skin sensitization; it evaluated whether a test substance sensitizes skin at the keratinocyte or *in vivo* levels (OECD, 2018a; OECD, 2018b). We had previously confirmed the applicability of this method using metal oxide nanoparticles, and this study investigated the effect of dissolution of metal nanomaterials on skin sensitization (Kim et al., 2020).

The level of reproducibility of the predictions expected from the KeratinoSens^TM^ assay was approximately 85% within and across laboratories. The accuracy of identifying a skin sensitizer by this test method has been demonstrated to be 77% (155/201), with a sensitivity of 78% (71/91) (Natsch et al., 2013; EURL-ECVAM, 2014). Collectively, available data indicated the KeratinoSens^TM^ assay to be useful for identifying the risk of skin sensitization by various compounds (Emter et al., 2010; Andreas et al., 2011; EURL-ECVAM, 2014). After 145 chemical tests, the KeratinoSens^TM^ assay was recognized as an OECD guideline and is being evaluated as an alternative test guideline (Natsch et al., 2013). Skin sensitization tests are performed using purified chemicals; however, evaluation of materials that are not completely dissolved has also been reported (Andres et al., 2013; Settivari et al., 2015).

LLNA: BrdU-FCM assay was developed to replace the traditional radioisotopic LLNA; it could reduce the pain inflicted on animals, since it did not use immunoadjuvants and required a lesser number of animals for the test (OECD, 2018b). Skin sensitization study of some NPs such as TiO_2_ and ZnO had been performed previously using LLNA assay (Park et al., 2011; Jang et al., 2012); these were determined to be non-sensitizers.

To accurately identify the toxicity of nanomaterials, it would be important to produce a stable and uniform dispersion, since nanomaterial aggregates may exert different biological effects compared to well-dispersed nanomaterials (Bihari et al., 2008). The NPs show large aggregation when preparing suspensions with ordinary vehicle solutions in LLNA: BrdU-FCM assays. According to Bihari et al., 2008; Lee et al., 2016, the use of inactivated serum of the same species/strain as a dispersant could contribute to the reduction of hydrodynamic size (reduction of aggregation) in the NPs suspension.

In our study, the same substance was evaluated using the two test methods. It was found that TiO_2_ NP does not exhibit skin sensitization when evaluated using skin sensitization assay. Nickel is known as a positive sensitizer in humans and as per GMPT test results. However, as a limitation, nickel is shown as ‘false negative’ in the alternative test method (Williams et al., 2015). In the KeratinoSens^TM^ assay, CoO and CuO NPs were positive sensitizers while they were negative in *in vivo* results. Copper and cobalt are reported as sensitizing substances (Wang et al., 2018); however, in this study, the two test methods showed contradictory results.

Factors such as “very small,” “skin injury,” or “solubility of the materials” can promote the penetration of the substance into the skin. Skin penetration is possible at sizes below 20 nm, but nanomaterials are known to have low skin penetration (Filon et al., 2015; Yoshioka et al., 2017). When a sensitizing substance does not pass through the skin, accurate test results may not be obtained. In this study, it was confirmed that nanomaterials form large aggregates in the stratum corneum of the ear tissue. In addition, since the size of the nanomaterials of the working fluid used in the *in vivo* test is 300∼500 nm or more, it is considered that it could not have penetrated the skin.

We estimated that the difference in the *in vivo* and *in vitro* results of CuO and CoO NPs was due to their ability to generate metal ions. CuO and CoO NPs used in this test are reported to exhibit high solubility in the intracellular environment (Jeong et al., 2018). These highly ionized NPs rapidly dissolve in the lysosome and are released like a “Trojan horse,” causing cytotoxicity. The results of the metal ions treated with the same ion concentration as the CoO and CuO NPs showed a remarkably similar concentration-dependent pattern in the KeratinoSens^TM^ assay. When looking at the results of Co_3_O_4_ NPs, which has the same element as cobalt but does not dissolve well, CoO NPs showed more similarity with cobalt ions. In addition, the chelation test results of metal ions proved that the removal of metal ions does not induce sensitization. Therefore, the result obtained through this study is that the dissolution of metal NMs had an important effect on inducing skin sensitization. Our study investigated the potential of skin sensitization for five metal oxide NPs. Since only a few of the various NMs have been evaluated, more studies are needed to elucidate the mechanism of in-depth skin sensitization by NMs.

## Conclusion

In this study, evaluation of skin sensitization by NPs, through two skin sensitization test methods, showed the sensitization potential of CuO and CoO NPs. The effect was induced by the constituent elements of fast-dissolving NPs. Based on ion chelation data; metal ion release was confirmed as the major “factor” for skin sensitization. If the NPs are to be applied to alternative test methods or investigating skin sensitization, their physicochemical properties, including dissolution of NPs, would need to be considered. However, further investigations would be required to elucidate the mechanism underlying NPs-induced skin sensitization.

## Data Availability

The original contributions presented in the study are included in the article/Supplementary Material, further inquiries can be directed to the corresponding authors.
