# Corrigendum: Copper and Cobalt Ions Released From Metal Oxide Nanoparticles Trigger Skin Sensitization

**DOI:** 10.3389/fphar.2021.670581

**Published:** 2021-04-14

**Authors:** Sung-Hyun Kim, Jin Hee Lee, Kikyung Jung, Jun-Young Yang, Hyo-Sook Shin, Jeong Pyo Lee, Jayoung Jeong, Jae-Ho Oh, Jong Kwon Lee

**Affiliations:** Division of Toxicological Research, National Institute of Food and Drug Safety Evaluation, Ministry of Food and Drug Safety, Osong, South Korea

**Keywords:** skin sensitization, alternative test, KeratinoSens TM, LLNA, dissolving nanoparticles, nanoparticles, copper, cobalt

In the original article, there was an error. The value 0.00 μM was mistakenly inserted instead of 316.57 μM.

A correction has been made to **Results*,* Evaluation of NPs-Induced Sensitization in the KeratinoSens** ^**TM**^ **Assay, Paragraph 1**:

“The five metal oxide NPs were assessed for their skin sensitization potential using the KeratinoSens™ assay; the data are shown in Table 2 and Figure 2. CuO and CoO NPsinduced activity of the luciferase reporter by over 1.5-fold, suggesting their ability to cause skin sensitization. The other NPs did not increase luciferase activity in the KeratinoSens™ assay. The EC_1.5_ value for CuO and CoO NPs was 1.38 and 316.57 μM respectively, classifying them as sensitizers, whereas the values were >1,000 μM for the remaining NPs, classifying them as non-sensitizers.”

The authors apologize for this error and state that this does not change the scientific conclusions of the article in any way. The original article has been updated.

